# Reforming Cardiovascular Care in the United States towards High-Quality Care at Lower Cost with Examples from Model Programs in the State of Michigan

**DOI:** 10.5041/RMMJ.10151

**Published:** 2014-07-25

**Authors:** Daniel Alyeshmerni, James B. Froehlich, Jack Lewin, Kim A. Eagle

**Affiliations:** 1 Department of Cardiovascular Diseases, University of Michigan Health System, Ann Arbor, MI, USA; and; 2 Cardiovascular Research Foundation, New York, NY, USA

**Keywords:** Affordable Care Act, cardiovascular medicine, health care costs, health care reform, quality improvement

## Abstract

Despite its status as a world leader in treatment innovation and medical education, a quality chasm exists in American health care. Care fragmentation and poor coordination contribute to expensive care with highly variable quality in the United States. The rising costs of health care since 1990 have had a huge impact on individuals, families, businesses, the federal and state governments, and the national budget deficit. The passage of the Affordable Care Act represents a large shift in how health care is financed and delivered in the United States. The objective of this review is to describe some of the economic and social forces driving health care reform, provide an overview of the Patient Protection and Affordable Care Act (ACA), and review model cardiovascular quality improvement programs underway in the state of Michigan. As health care reorganization occurs at the federal level, local and regional efforts can serve as models to accelerate improvement toward achieving better population health and better care at lower cost. Model programs in Michigan have achieved this goal in cardiovascular care through the systematic application of evidence-based care, the utilization of regional quality improvement collaboratives, community-based childhood wellness promotion, and medical device-based competitive bidding strategies. These efforts are examples of the direction cardiovascular care delivery will need to move in this era of the Affordable Care Act.

## INTRODUCTION

Advanced technology and infrastructure, a superbly trained work force, and excellent academic institutions characterize the US health care system. Many believe it is the world leader in science, medical education, and health innovation as evidenced by the fact that immigrant physicians account for 27% of trainees in the United States and a quarter of the physician work force in the United States.^1^ It is estimated that approximately 84% of Americans have public or private health insurance.^2^ Unfortunately, the remaining 16% of Americans are either underinsured or uninsured. A quality chasm exists in American health care. Care fragmentation and poor coordination contribute to expensive care with highly variable quality in the United States. It is estimated that in 2011 the United States spent $2.7 trillion dollars on health care. If health care spending is not curbed, it is estimated that by 2020 spending may be as much as $4.6 trillion dollars.^3^

The passage of the Patient Protection and Affordable Care Act (ACA) in 2010 represents a large shift in how health care is financed and delivered in the United States. The objective of this review is to describe some of the economic and social forces driving health care reform, provide an overview of the ACA, and review specific programs underway in the state of Michigan aimed at improving the quality and reducing the cost of cardiovascular care.

## THE US HEALTH CARE LANDSCAPE

In 2014, the US economy is facing many political challenges as it continues to emerge from recession. One of these challenges is the expiration of a portion of the Bush era tax cuts that have increased income taxes on the highest earners in the United States. Other challenges are the need to re-raise the national debt ceiling and the potential cuts in physician reimbursement associated with the sustainable growth rate. All of these pressures are compounded by a health care system that is spending out of control and growing faster than the national gross domestic product.^4^

There are a number of opportunities to reduce health care spending in the United States. Excess care is thought to be responsible for $750 billion, medical errors account for $50 billion, and defensive medicine accounts for approximately $50 billion.^5^ The annual rate of malpractice litigation affects an estimated 8% of cardiologists and as many as 20% of cardiovascular surgeons.^6^

### High Per Capita Health Care Expenditures without a Correlated Improvement in Outcomes

**Figure 1.**
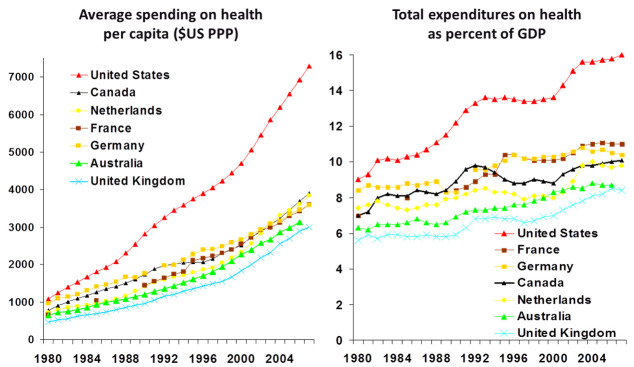
**International Comparison of Spending on Health, 1980–2007.** PPP; purchasing power parity. Taken from The Commonwealth Fund website, with permission; http://tinyurl.com/n2hvucv. Last accessed June 2014.

The United States spends far more than other Western societies that have excellent health care. By 2012 WHO estimates, the United States spent $8,607 per capita on health care; this is the most in the world and close to two thousand dollars more than the second highest spender, Luxembourg.^7^ It is projected that if costs are not curbed, as much as 19.8% of the per capita dollars in the United States will be spent on health care by 2020. Figure 1 shows an international comparison of annual spending on health per capita or total expenditures on health as a percent of the gross domestic product from 1980 to 2007.

**Figure 2.**
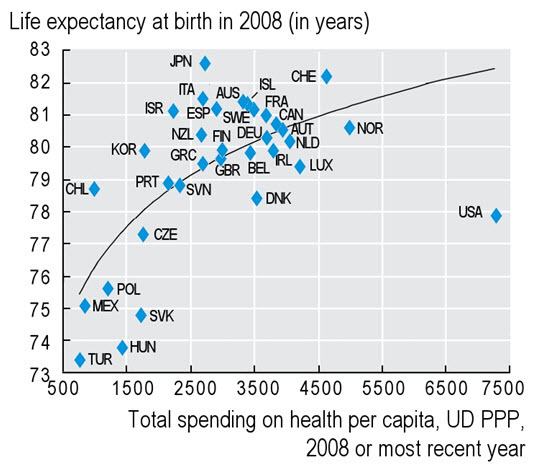
**International Comparison of Life Expectancy versus Health Care Spending Per Capita.** Reused with permission from OECD (2011), “Health spending,” in *Society at a Glance 2011: OECD Social Indicators*, OECD Publishing; http://dx.doi.org/10.1787/soc_glance-2011-en. Last accessed June 2014. Abbreviations: AUS, Australia; AUT, Austria; BEL, Belgium; CAN, Canada; CHE, Switzerland; CHL, Chile; CZE, Czech Republic; DEU, Germany; DNK, Denmark; ESP, Spain; FIN, Finland; FRA, France; GBR, United Kingdom; GRC, Greece; HUN, Hungary; IRL, Ireland; ISL, Iceland; ISR, Israel; ITA, Italy; JPN, Japan; KOR, Korea; LUX, Luxembourg; MEX, Mexico; NLD, Netherlands; NOR, Norway; NZL, New Zealand; POL, Poland; PRT, Portugal; SVK, Slovak Republic; SVN, Slovenia; SWE, Sweden; TUR, Turkey; USA, United States; UD PPP; United States dollars purchasing power parity.

The troubling fact about health care spending in the United States is that it does not correlate with better outcomes or population health. The average life expectancy in the United States is 78.4 years, ranking fiftieth overall when compared with 221 nations.^8^ Figure 2 illustrates that the United States is an outlier with regard to life expectancy and health care spending per capita. This is despite spending two and sometimes three times more on health care than other developed countries.

### Health Care Spending’s Disproportionate Impact on the Deficit

**Figure 3.**
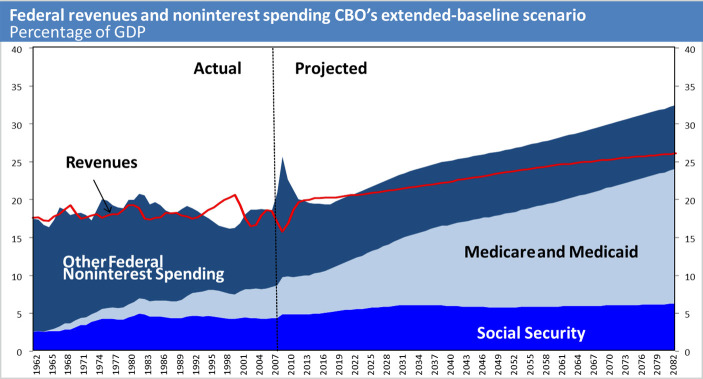
**The Congressional Budget Office’s 2009 Projection of Federal Revenues and Non-interest Spending**. Modified from the Congressional Budget Office, Long-Term Budget Outlook, Figure 1-1; June 2009; http://www.cbo.gov/publication/20776. Last accessed June 2014.

With regard to deficit spending, the Medicare and Medicaid entitlement programs represent by far the most important factors affecting the revenue expense balance for the US government. Figure 3 illustrates the potential deficit between revenues and spending of government entitlement programs and demonstrates that earnest reductions in deficit spending must focus on Medicare and Medicaid. Runaway health care spending without social security reform will force the government to continue deficit spending and increase the economic strain on the next generation.

### Challenges with Health Care Access

Comparing similar groups, the US insured population is similar to Canadians in terms of overall health care activity, having a regular physician, visiting a physician within a year, having unmet health needs, and overall affordability of medications.^9^ Conversely, the uninsured population in the United States lags far behind these groups and often lacks a regular doctor, has not seen a doctor within the year, describes unmet health needs, and cannot afford medicines (Table 1).

**Table 1.** Access to Health Care: Canada and the United States.

	Canada	United States	

		Insured	Uninsured
Have regular physician	85%	85%	40%
Seen MD within year	83%	86%	56%
Unmet health needs?	11%	10%	36%
Can’t afford medications	5%	8%	28%

Data from Lasser, Himmelstein, and Woodhandler, Access to care, health status, and health disparities in the United States and Canada. *Am J Public Health* 2006;96(7):1300–7.

### The Financial Burden of Health Care Costs

The impact of the financial burden of health care on individuals and small businesses in the United States often works against economic growth. It is estimated that more than 60% of personal bankruptcies in the United States arise from the inability to pay the costs of essential health care for serious illness.^10^ The financial burden health care costs can have is also responsible for the failure of a significant number of small businesses.

The rising costs of health care since 1990 have had a huge impact on individuals, families, businesses, the federal and state governments, and the national deficit. Poor access and fragmentation of care add further strain to the US health care system. This confluence of social and economic forces was the impetus behind the Affordable Care Act.

## THE PATIENT PROTECTION AND AFFORDABLE CARE ACT

The ACA is a public/private reform law that originated in the United States House of Representatives and was championed by the Obama Administration. It narrowly passed both Congressional houses and was signed into law by President Obama in 2010. The Affordable Care Act allows for 35 million uninsured Americans to obtain insurance and creates reforms of private insurance rules and Medicare and Medicaid services. It allocates investments in prevention and public health, comparative effectiveness research, and tests new care delivery models by establishing the Centers for Medicare and Medication Innovation. Through its significant impact on payment reform, it encourages integration of doctors and hospitals and the formation of shared-risk accountable care organizations that will be responsible for managing population health. The ACA is composed of nine independent titles, and an abbreviated description of all of these titles can be found in Table 2.

**Table 2.** The Patient Protection and Affordable Care Act.

Title	Description
I	Focuses on insurance reform, and establishes access to discounted insurance exchanges in all states, covers children through the age of 26 on their parent’s insurance, and increases tax penalties both for individuals and businesses that do not have or provide insurance.
II	Focuses on Medicaid and creates a guarantee of public or private insurance for lower-income individuals and families. It provides the choice of either public or private plans on the insurance exchanges and allows individuals without employer insurance of higher income to use the exchanges.
III	Focuses on Medicare care delivery reform. It establishes a national quality strategy, endorses and invests in Accountable Care Organizations under the Centers for Medicare & Medicaid Services (CMS), creates incentives for preventing hospital infections and readmissions, establishes state and federal high-risk pools for insurable patients, and invests in Medicare hospital value-based purchasing. It also creates the Center for Medicare and Medicaid Innovation where new delivery models are tested through public–private partnerships and challenge grants. This piece of the legislation creates and further develops Medicare’s quality measure and reporting programs as well as programs in comparative effectiveness research in quality of care, safety, and cost containment.
IV	Focuses on prevention and outlines a national prevention strategy, the creation of the national prevention trust fund, a requirement that certain restaurants and vending machines provide nutrition information, and invests in state governments and work places to promote wellness in the work place.
V	Focuses on health professions education grants and incentivizing primary care for physicians, nurses, and mid-level practitioners.
VI	Focuses on fraud and abuse as well as nursing home transparency. This includes the Physician Sunshine Act that requires physicians to disclose all money received from pharmaceutical and device industries and creates a national database for this purpose.
VII	Focuses on the FDA rules regarding biological or bio-similar drugs.
VIII	Provides Americans with a new option to finance long-term care services in the event of a disability.
IX	Relates to revenue, including a FICA tax on payroll that will help to paypartially for some of the new services associated with the ACA. In addition there will be new fines on businesses and individuals for lack of coverage.

### Partisan Contention over the Affordable Care Act

The Affordable Care Act has been the source of considerable partisan contention in the United States, and disagreements over its funding in the Congressional budget even resulted in a two-week partial government shutdown in October 2013. The law was challenged by the National Federation of Independent Business in the highest court in the United States, the Supreme Court, and was ultimately upheld in 2012 with an amendment to the Medicaid expansion provision.

The challenges facing the Affordable Care Act in 2014 are mainly political and operational. The ideas in the legislation must translate into impactful changes that improve population health and deliver better care at lower cost, or the law could fall prey to partisan impediments and disagreements.

### The Affordable Care Act in 2014

In 2014, two centerpieces of the legislation go into effect. The health insurance mandate requires people to obtain insurance or pay a penalty, which is legally enforceable as a tax. Further, previously uninsured people now either qualify for Medicaid or are able to purchase plans on health insurance exchanges. As part of the Supreme Court ruling in 2012, states are no longer required but rather have the option to expand Medicaid. Twenty-five states and the District of Columbia have elected to expand Medicaid in order to cover the uninsured. Michigan has chosen to expand Medicaid and is one of seven states with a Republican administration that havechosen this route. Given its mixed legislature, Michigan is uniquely positioned to be a model for bipartisan health care reform.^11^

### The Creation of Health Insurance Exchanges

States have the option to build their own health insurance exchanges or utilize the federal exchange “healthcare.gov.” The rollout of the federal insurance exchange was riddled with logistic and technological difficulties and was the source of considerable bad publicity for the ACA. With improvements in the federal exchange, enrollment numbers initially lagged behind projections but have approached expectations with 4.2 million Americans signing up for health insurance by using the exchanges as of February 2014.^12^ In the coming year, insurance companies will enter a competitive race in the exchanges, and medical care delivery systems will be reorganized to demonstrate performance, reduce costs, and create more accountability around quality, safety, prevention, efficiency, and appropriateness of care.^13^

The blueprint for the Affordable Care Act, the Massachusetts health reform effort that began in 2006, has already led to improvements in the health status of Massachusetts’ residents. When compared to rates before health reform implementation, the proportion of residents receiving preventive services is higher in Massachusetts than in neighboring New England states.^14^

The ACA enactment in the United States will significantly impact the US health care system and has the potential to transform care delivery. However, a hallmark of the law is that although a substantial amount of federal aid is used to support reforms, it allows for a considerable portion of reform and improvement to occur at the local and state level. The next portion of this review focuses on regional efforts in Michigan aimed at improving cardiovascular care and reducing cost that can serve as models for other similar projects. All of these programs pursue the popularized Institute for Healthcare Improvement’s “Triple Aim” of improving the experience of care, improving the health of populations, and reducing per capita costs of health care.^15^

## EXAMPLES OF CARDIOVASCULAR CARE QUALITY IMPROVEMENT IN THE STATE OF MICHIGAN

As health care reorganization occurs at the federal level, local and regional efforts can serve as models that can accelerate improvement toward achieving better population health and better care at lower cost. In the paragraphs below we describe several initiatives in Michigan that can serve as examples of the direction cardiovascular care delivery will need to move in this new era of the Affordable Care Act. These programs pursue better health outcomes at lower cost by the application of better evidence-based care, the utilization of regional quality improvement collaboratives, community-based childhood wellness promotion, and medical device-based competitive bidding strategies.

**Figure 4.**
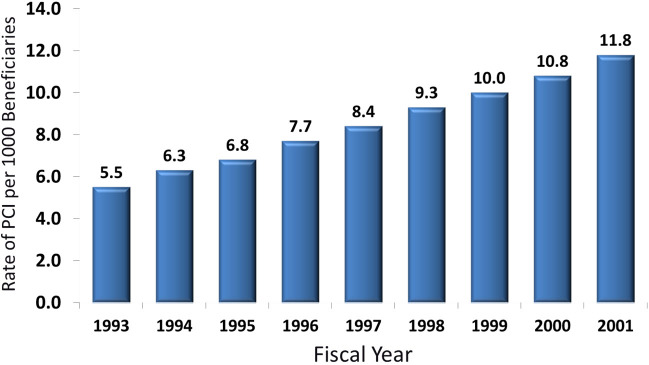
**Temporal Changes in Rates of Cardiac Percutaneous Coronary Intervention (PCI) per 1000.** Medicare beneficiaries 1993–2001, adjusted for age, gender, and race. Modified from Table 2 of Lucas et al.,^16^ with permission.

**Figure 5.**
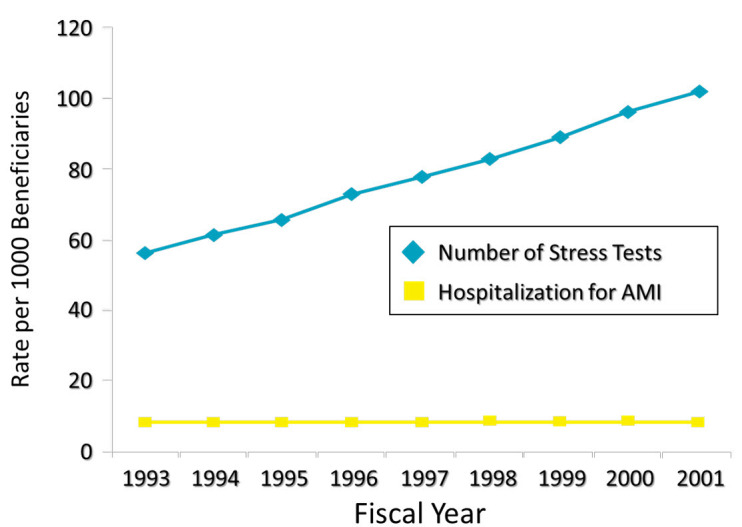
**Trends in Population-based Hospitalization for AMI and Stress Testing among Medicare Beneficiaries 1993-2001, Adjusted for Age, Gender, and Race.** Modified from Table 2 of Lucas et al.,^16^ with permission.

Over the past 15–20 years, it has been apparent that cardiovascular care expenditures are proportionally higher than the benefit in cardiovascular risk reduction in the United States. Perhaps one of the most obvious areas is the use of cardiac imaging stress tests and coronary artery angioplasty (Figures 4 and 5). Despite the steadily increasing use of stress testing and percutaneous coronary intervention in stable coronary disease, there has been no impact on hospitalization for acute heart attack.^16^ The COURAGE trial and other similar investigations have proven that, with excellent medical therapy, the routine use of stress testing and percutaneous coronary intervention for stable coronary disease has no impact on either survival or acute MI.^17^ In the United States, the “weaning” from this financial stream related to routine stress testing followed by elective coronary intervention is ongoing and should result in substantial savings.^18^

### A Systematic Approach to the Application of Evidence-Based Care to Improve Outcomes and Cost

Recognizing this area for improvement, teams at the University of Michigan have focused on applying the perioperative cardiac care guidelines to patients being considered for elective aortic surgery.^19^ As outlined in Table 3, by implementing a national guideline that carefully outlines the indications for stress testing, coronary angiography, and coronary intervention prior to elective aortic aneurysm repair, caregivers both reduced cost and improved outcomes through the use of evidence-based practice guidelines. A focus on implementing evidence-based care strategies will allow the US health care system to achieve better outcomes for its expenditures.

**Table 3.** Resource Use and Outcomes after Implementation of American College of Cardiology/American Heart Association Preoperative Risk Assessment Guidelines.

Resource Utilization	I Controls *n*=102	II Post Guideline *n*=94	III Late Post Guideline *n*=104	*P* Value	

				I vs II	I vs III
Stress test	90 (88%)	44 (47%)	43 (41%)	<0.001	<0.001
Coronary Angiography	24 (24%)	10 (11%)	11 (11%)	<0.05	0.01
PTCA or CABG	24 (24%)	2 (2%)	6 (6%)	<0.001	<0.001
Length of stay (days)	20.7	13.2		<0.001	
Preop cost	$1087	$171		<0.001	
Cost per case	$21,947	$15,188		0.02	
Outcomes:					
Death	4 (4%)	3 (3%)	0 (0%)	0.77	
MI	7 (7%)	3 (3%)	5 (5%)	0.24	
Death or MI	11 (11%)	4 (4%)	5 (5%)	0.08	

PTCA, percutaneous transluminal coronary angioplasty; CABG, coronary artery bypass grafting.

**Figure 6.**
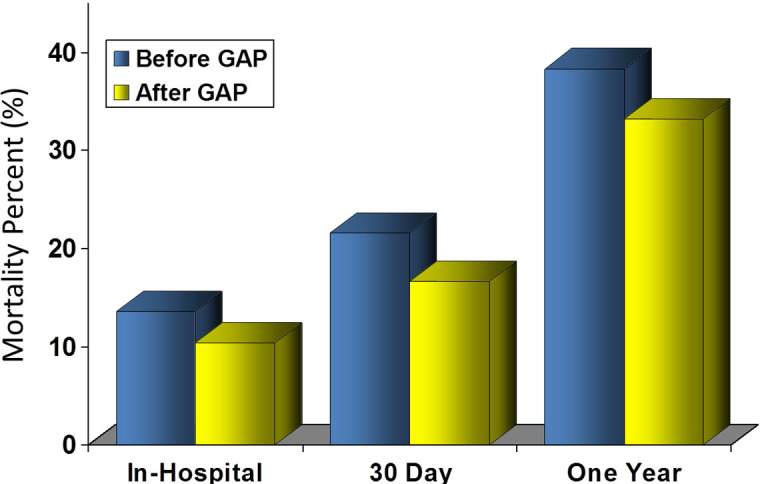
**American College of Cardiology Guidelines in Applied Practice for Acute Myocardial Infarction (ACC GAP AMI) Program Mortality Benefit in Medicare Patients.** From Table 4 of Eagle et al.,^22^ with permission.

By improving the systems through which evidence-based care is delivered, the Guideline Applied in Practice project in Michigan improved post-MI care over a 10-year period. By involving 400 cardiologists, 33 hospitals, and teams of physician and nurse leaders, the Guideline Applied in Practice project proved that embedded reminders into care processes aimed at the effective use of early aspirin, beta blocker and LDL measurement, ACE inhibitors, smoking counseling, cholesterol treatment, and dietary counseling improved the performance measures during acute coronary syndrome care.^20^–^22^ Most importantly, the study demonstrates this practice has a favorable impact on mortality after MI in Medicare beneficiaries, lowering the in-hospital mortality from 13.6% to 10.4%, and the one-year mortality from 38.3% to 33.2% (Figure 6). This effort illustrates that by measuring care on a regular basis, interventions can be identified to improve systems and quality in a sequential and iterative basis through rapid-cycle quality improvement. The ability to identify defects and rapidly improve outcomes will allow health systems to respond with agility in the new health care environment.

### Utilizing Regional Quality Improvement Collaboratives

**Figure 7.**
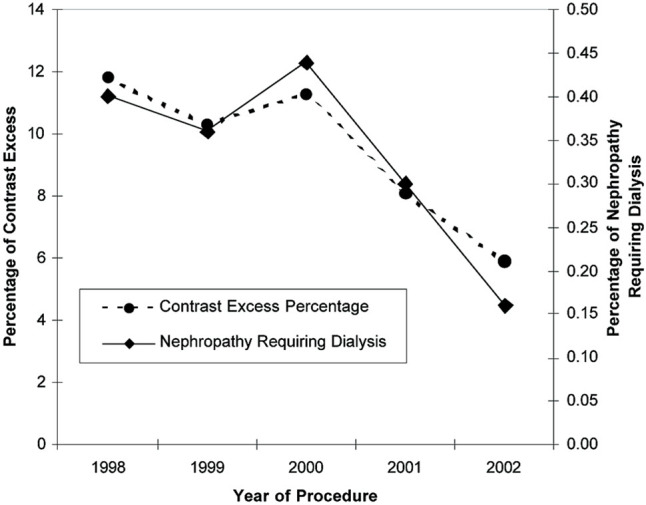
**Percentage of Patients Exceeding the Maximum Weight and Creatinine-adjusted Contrast Dose, and Percentage of Patients Developing Nephropathy Requiring Dialysis.** From Figure 2 of Moscucci et al.,^26^ with permission.

A similar approach is taken at a larger scale in The Blue Cross Blue Shield of Michigan Cardiovascular Collaborative (BMC2) quality improvement collaborative.^23^,^24^ In this program, all process and outcome measures of the state’s coronary interventions are documented and entered in a central core laboratory that produces regular reports for operators and hospitals. Through their efforts, the BMC2 investigators identified a maximum allowable contrast dose for patients undergoing an angioplasty that predicted a 6-fold risk increase in the development of acute renal failure.^25^ By implementing a strategy where the maximum allowable contrast dose was calculated before a patient underwent angioplasty in each hospital, the investigators were able to achieve a significant reduction in the frequency of dialysis-dependent renal failure after coronary intervention (Figure 7).^26^ This is an example of the detection and prevention of a relatively infrequent but severe complication related to care processes that would have gone undetected without a large collaborative study. This model in Michigan can be readily duplicated by other states, and the Affordable Care Act is likely to lead to similar large regional and national consortia for improving care and preventing harm.

### Investing in Community-Based Health and Wellness Promotion

By emphasizing prevention and investing in its community, the University of Michigan has been successful in improving the health status of children across the state. The Project Healthy Schools initiative in Michigan targets middle schools with an intervention to improve childhood health. Eagle and colleagues have demonstrated through a number of studies that by developing an educational curriculum in middle schools, improving cafeteria offerings, and changing beverages and snacks sold in vending machines to healthier choices, the health status of middle school children can improve.^27^ In the Michigan experience, this improvement is seen within 10 weeks of the intervention and is sustained over a period of three years.^28^ The fast food and electronic gaming revolution has created a generation of young people who are overweight, unfit, and much more likely to develop hypertension, diabetes, and coronary disease later in life. Project Healthy Schools is one example where investing in community health has the opportunity to prevent long-term adverse cardiac events. This partnership with communities can be easily modeled by other health systems in this era of health reform.

### Reducing Health Care Costs through Competitive Bidding Strategies for Medical Devices

**Figure 8.**
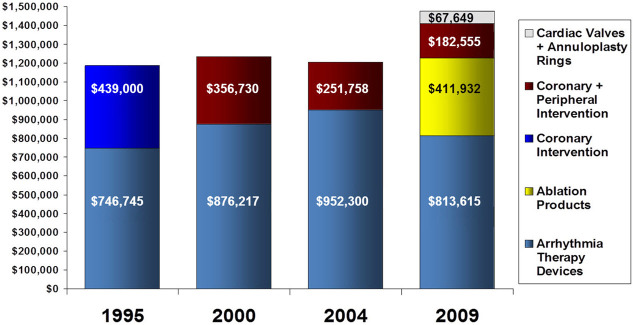
**Estimated Annualized Savings Gained through Combining Negotiation Strategies and Additional Product Lines.** Modified from Figure 1, Keast et al.,^32^ with permission.

Emanuel and colleagues have written that transparency in the cost of care and a focus on improving supply line costs have the potential to reduce the cost of care delivery through competitive bidding strategies.^29^ In cardiovascular care delivery, pacemakers, defibrillators, coronary catheters, stents, and cardiac valves are a remarkable source of cost. At the University of Michigan, the interventional teams in cardiac surgery, electrophysiology, and coronary intervention have been able to show a substantial reduction in costs through a series of competitive bidding strategies with vendors of various cardiovascular products.^30^–^33^ Figure 8 shows the analyzed savings gained through negotiation strategies in various cardiovascular product lines over a period of 10 years.

## Conclusion

The future of effective, safe, timely, and patient-centered cardiovascular care will only be created through the active participation of patients, physicians, nurses, staff, administrators, legislators, and the general public. In the United States, the Affordable Care Act is occurring at a time when the nation’s health care budget is unsustainable. Despite all the rancor and disagreement on the “how,” the authors are in general agreement on the need for reforms and believe the examples from Michigan provide numerous entry points for further exploration and development in Michigan and across the country. The US health care system of the future is currently being crafted and designed, and, as this occurs, openness, agility, and a focus on the needs of patients will make this transition smoother.

## Abbreviations:

ACA: Patient Protection and Affordable Care Act;
BMC2: the Blue Cross Blue Shield of Michigan Cardiovascular Collaborative.
